# Overcoming Intrinsic Doxorubicin Resistance in Melanoma by Anti-Angiogenic and Anti-Metastatic Effects of Liposomal Prednisolone Phosphate on Tumor Microenvironment

**DOI:** 10.3390/ijms21082968

**Published:** 2020-04-23

**Authors:** Emilia Licarete, Valentin Florian Rauca, Lavinia Luput, Denise Drotar, Ioana Stejerean, Laura Patras, Bogdan Dume, Vlad Alexandru Toma, Alina Porfire, Claudia Gherman, Alina Sesarman, Manuela Banciu

**Affiliations:** 1 Department of Molecular Biology and Biotechnology, Faculty of Biology and Geology, Babes-Bolyai University, 400006 Cluj-Napoca, Romania; emilia_licarete@yahoo.com (E.L.); valirauca@yahoo.co.uk (V.F.R.); lavinia_luk@yahoo.com (L.L.); deniseminervadrotar@gmail.com (D.D.); ioana.stejerean@yahoo.com (I.S.); patras.laura88@yahoo.com (L.P.); bobihas@gmail.com (B.D.); tomavlad91@yahoo.com (V.A.T.); manuela.banciu@ubbcluj.ro (M.B.); 2 Molecular Biology Centre, Institute for Interdisciplinary Research in Bio-Nano-Sciences, Babes-Bolyai University, 400271 Cluj-Napoca, Romania; 3 Centre of Systems Biology, Biodiversity and Bioresources, Faculty of Biology and Geology, Babes-Bolyai University, 400006 Cluj-Napoca, Romania; 4 Institute of Biological Research, 400015 Cluj-Napoca, Romania; 5 National Institute for Research and Development of Isotopic and Molecular Technologies, 400293 Cluj-Napoca, Romania; 6 Department of Pharmaceutical Technology and Biopharmaceutics, Faculty of Pharmacy, Iuliu Hatieganu University of Medicine and Pharmacy, 400012 Cluj-Napoca, Romania; alinatuns@yahoo.com; 7 Department of Functional Genomics and Experimental Pathology, The Oncology Institute “Prof. Dr. Ion Chiricuta”, 400015 Cluj-Napoca, Romania; ghermanclau@yahoo.com

**Keywords:** angiogenesis, combined therapy, liposomes, melanoma, tumor associated macrophages

## Abstract

Regardless of recent progress, melanoma is very difficult to treat, mainly due to the drug resistance modulated by tumor cells as well as by the tumor microenvironment (TME). Among the immune cells recruited at the tumor site, tumor associated macrophages (TAMs) are the most abundant, promoting important tumorigenic processes: angiogenesis, inflammation and invasiveness. Furthermore, it has been shown that TAMs are involved in mediating the drug resistance of melanoma cells. Thus, in the present study, we used liposomal formulation of prednisolone disodium phosphate (LCL-PLP) to inhibit the protumor function of TAMs with the aim to sensitize the melanoma cells to the cytotoxic drug doxorubicin (DOX) to which human melanoma has intrinsic resistance. Consequently, we evaluated the in vivo effects of the concomitant administration of LCL-PLP and liposomal formulation of DOX (LCL-DOX) on B16.F10 melanoma growth and on the production of key molecular markers for tumor development. Our results demonstrated that the concomitant administration of LCL-PLP and LCL-DOX induced a strong inhibition of tumor growth, primarily by inhibiting TAMs-mediated angiogenesis as well as the tumor production of MMP-2 and AP-1. Moreover, our data suggested that the combined therapy also affected TME as the number of infiltrated macrophages in melanoma microenvironment was reduced significantly.

## 1. Introduction

Melanoma is the most aggressive skin cancer and very difficult to treat in advanced stages. Furthermore, epidemiologic data suggested a continuous increase in incidence during the last decades [1]. Metastatic melanoma has a poor prognosis, mainly due to the tumor aggressiveness and rapid development of cancer cell resistance to both chemotherapy and immunotherapy, as well to the new generation tumor-targeted drugs [2]. Moreover, clinical and experimental data have demonstrated the critical role of the tumor microenvironment (TME) in the settlement of drug resistance of cancer cells as well as in inducing the immune non-responsiveness at the tumor site [3,4]. Primarily, tumor cells induced a proneoplastic TME via the creation of an immunosuppressive niche by the selective recruitment of regulatory T cells and the polarization of the tumor-associated macrophages (TAMs) to an alternative, protumor phenotype (M2 macrophages) [5,6,7]. Furthermore, among all immune cells present in tumors, TAMs are the key cell players which are involved not only in the preservation of the immunosuppressive state of the TME, but also in promoting tumor progression via the modulation of essential tumorigenic processes such as: angiogenesis, drug resistance, and metastasis [8,9]. It should be noted that alternatively activated TAMs can protect tumor cells against the cytotoxicity induced by chemotherapeutic drugs [10]. Therefore, TAMs-targeted treatments could be used to improve therapeutic strategies for melanoma when they are used in combination with cytotoxic drugs. In this view, our previous study has shown that TAMs-targeted therapy based on prednisolone disodium phosphate (PLP) encapsulated in long circulating liposomes (LCL) (LCL-PLP) exerted strong antitumor activity in B16.F10 melanoma-bearing mice via the inhibition of the proangiogenic function of intratumor macrophages [11]. Therefore, administration of the TAMs-targeted treatment could be a promising strategy to overcome the melanoma cell resistance to the specific cytotoxic drugs. To demonstrate the role of TAMs-targeted therapy as sensitizer of melanoma cells to the antitumor agent, doxorubicin (DOX) was selected as a consequence of its inefficacy in treating human melanoma, mainly as a result of the intrinsic resistance of this cancer type to this drug [12,13]. Moreover, our recent research has gained some evidence regarding the potential of PLP to improve DOX cytotoxicity on B16.F10 murine melanoma cells in vitro via the inhibition of the proangiogenic function of TAMs [14]. Based on these previous findings, we investigated whether PLP can sensitize B16.F10 melanoma tumors in vivo to the treatment with DOX when both antitumor agents are administered intravenously in mice as encapsulated forms in LCL. LCL ensured the passive accumulation of both drugs in tumors, due to the enhanced permeability of tumor vasculature as compared to healthy endothelium [15]. Our data suggested that concomitant administration of LCL-PLP and LCL-DOX induced strong antitumor actions. Thus, the combined therapy induced a strong inhibition of tumor growth mainly via the anti-angiogenic actions as well as inhibitory effects on two important proteins involved in tumor progression and metastasis (MMP-2 and AP-1). In tight connection with the anti-angiogenic action of the combined liposomal drug therapy on B16.F10 melanoma in vivo, our data suggested that the combined therapy also affected TME as the number of infiltrated macrophages in melanoma microenvironment was reduced significantly.

## 2. Results

### 2.1. Effects of the Liposomal Combination Therapy on the Proliferation of B16.F10 Cell Co-Cultivated with Murine Macrophages

**Figure 1:**
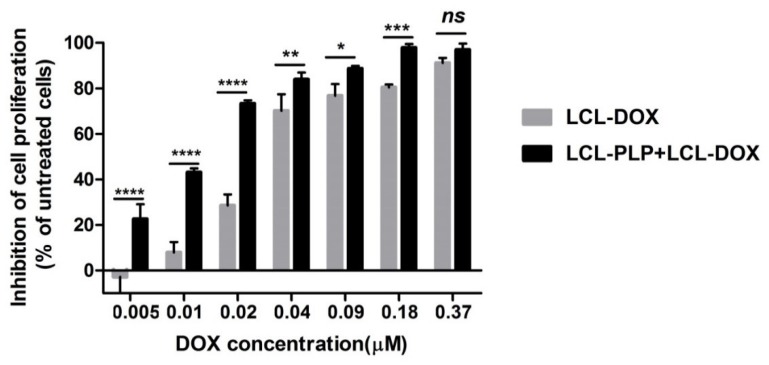
Effects of LCL-PLP + LCL-DOX on B16.F10 cell proliferation. B16.F10 mouse melanoma cells co-cultured with murine macrophages were incubated with solutions of different concentrations of DOX (ranging from 0.005–0.37 µM) encapsulated in LCL (LCL-DOX) in the presence as well as in the absence of 410 μM PLP as LCL-PLP for 48 h. LCL-DOX, LCL-DOX-treated cells; LCL-PLP + LCL-DOX, cells incubated with solutions of different concentrations of LCL-DOX and 410 μM LCL-PLP. Data are shown as mean ± SD of three measurements and represented as percentages of proliferation inhibition compared with the proliferation of control cells. (ns, not significant (*p* > 0.05); * *p* < 0.05; ** *p* < 0.01; *** *p* < 0.001; **** *p* < 0.0001).

To investigate whether LCL-PLP could potentiate the cytotoxicity of LCL-DOX on B16.F10 melanoma cells, we assessed the effects of LCL-DOX alone as well as in combination with liposomal PLP on the proliferation of these cancer cells co-cultured with macrophages. A proliferation ELISA test was based on the incorporation of BrdU into the DNA of the proliferating cells. The effects of the treatments were expressed as percentages of the inhibition of cell proliferation compared to the proliferation of the cells used as controls and shown in Figure 1. After 48 h of incubation, except for the highest concentration of LCL-DOX (0.37 μM) tested, the simultaneous administration of 410 μM PLP as a liposomal form with each DOX concentration in LCL significantly enhanced the antiproliferative effects of the cytotoxic drug on melanoma cells (Figure 1). At the highest concentration of DOX tested, the proliferation of B16.F10 cells was totally decelerated (over 90% inhibition of cell proliferation compared with control cancer cell proliferation) and therefore, the enhancing action of LCL-PLP on the cytotoxic drug effect on these cancer cells was overshadowed. These data are also supported by the IC50 value of DOX, that decreased three times when the cytotoxic drug was administered as combined liposomal drug therapy (0.011 µM) compared with IC50 value of DOX (0.033 µM), when LCL-DOX administration was not associated with LCL-PLP treatment.

### 2.2. The Combined Liposomal Drug Therapy Induced a Stronger Inhibition of the Melanoma Tumor Growth than Monotherapies Based on either LCL-DOX or LCL-PLP

**Figure 2:**
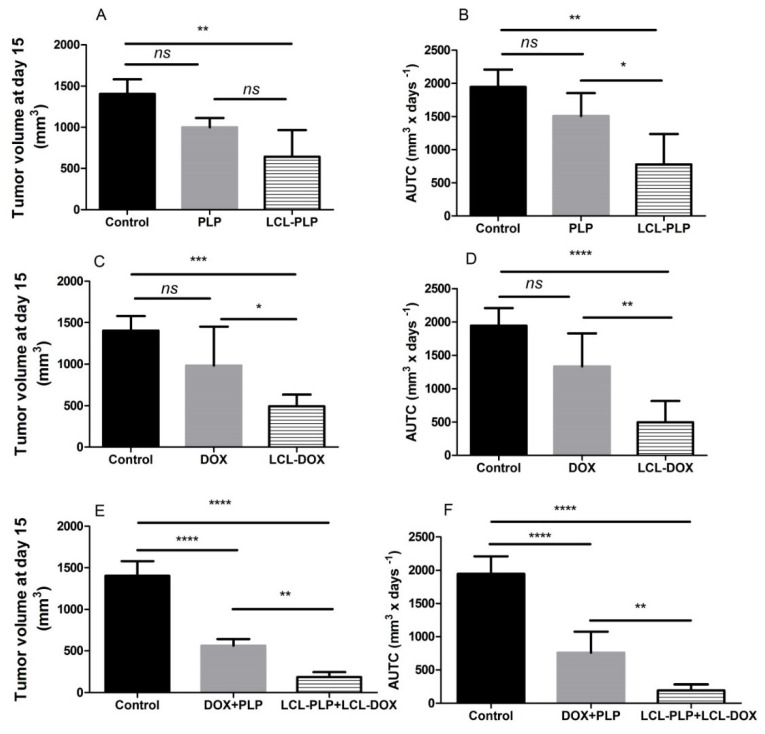
Effect of the LCL-PLP + LCL-DOX combined therapy on the B16.F10 melanoma growth in vivo. (**A**,**C**,**E**): for each experimental group, tumor volumes at day 15 after tumor cell inoculation were compared with the tumor volumes from control group measured at the same time point: (**B**,**D**,**F**): areas under the tumor growth curves (AUTC) until day 15. The results were expressed as mean ± SD of tumor volumes of five mice. ns—not significant (*p* > 0.05); * *p* < 0.05; ** *p* < 0.01; *** *p* < 0.001; **** *p* < 0.0001.

To assess whether the co-administration of LCL-PLP with LCL-DOX could potentiate the antitumor activity of cytotoxic drug encapsulated in LCL in B16.F10 melanoma-bearing mice, 10 mg/kg LCL-PLP and 5 mg/kg LCL-DOX were administered i.v simultaneously as well as alone at day 11 and 14 after tumor cell inoculation. The mice were sacrificed the following day, tumor tissue form each experimental group was collected and tissue lysates were obtained. The results were shown in Figure 2 and expressed as tumor volumes at day of sacrifice (Figure 2A,C,E) and areas under the tumor growth curves (AUTC) (Figure 2B,D,F). Our data suggested that the growth of B16.F10 melanoma in vivo was affected strongly after administration of each monotherapy based on either LCL-PLP (by 55–60%, *p* < 0.01) or LCL-DOX treatment (by 65–75%, *p* < 0.001) when compared with control tumors (untreated tumors or LCL-treated groups) growth according to tumor volumes measurements (Figure 2A,C) as well as AUTC data (Figure 2B,D). These antitumor activities were clearly enabled by the tumor-targeting properties of the liposomal formulations, since the same doses of either PLP or DOX administered alone as free forms did not show any inhibitory effects on melanoma growth (Figure 2A–D). Notably, both combined therapies affected the tumor growth, albeit with the higher degree for combined liposomal drug therapy compared to the administration of both free drugs (Figure 2E,F). Moreover, LCL-PLP + LCL-DOX was superior in terms of antitumor activity to both single liposomal drug therapies tested, inducing the almost total deceleration of the growth of B16.F10 melanoma tumors (by 87–90%, *p* < 0.0001) (Figure 2A–F). Therefore, the main mechanisms of the antitumor activity of LCL-PLP + LCL-DOX in B16.F10 murine melanoma-bearing mice were further investigated.

### 2.3. Liposomal Combination Therapy Induced Strong Anti-Angiogenic Actions on Melanoma in Vivo

**Figure 3:**
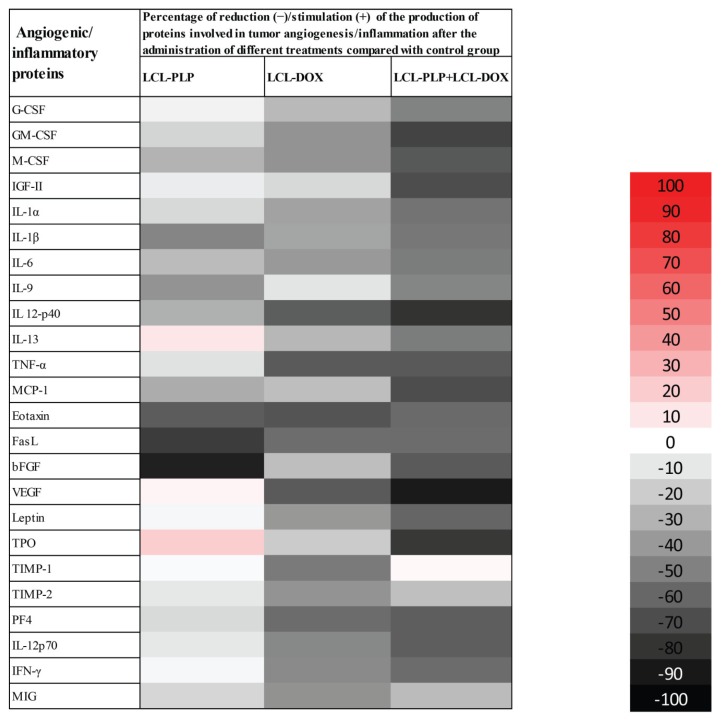
Effects of LCL-PLP + LCL-DOX combined therapy on the production of angiogenic proteins in melanoma tumors. Results are presented either as % of reduction (-) of tumor protein levels ranging from 0% (white) to 100% (black) or as % of stimulation (+) of production of proteins ranging from 0% (white) to 100% (red) in tumors after different treatments compared to levels of the same proteins in untreated tumors.

To evaluate the production of intratumor angiogenic and inflammatory proteins after administration of different liposomal treatments, we performed a screening for 24 angiogenic and inflammatory proteins in the tumor tissue lysates via protein array (RayBiotech Inc., Peachtree Corners, GA, USA) and results are shown in Figure 3 and Table 1. Tissue lysates were obtained from the tumor collected from each experimental group at the day of sacrifice (day 15 after tumor cell inoculation) after the i.v administration of each treatment at days 11 and 14 after tumor cell inoculation. LCL-PLP administered at 10 mg/kg induced a moderate (by 25–50%) reduction in the production of several pro-angiogenic proteins (M-CSF, IL-1β, IL-6, IL-9, IL-12p40, MCP-1). Other potent tumorigenic proteins such as eotaxin, bFGF, and FasL were strongly reduced (by 60–90%) after the treatment with LCL-PLP (Figure 3 and Table 1). Notably, 5 mg/kg LCL-DOX administered alone also exerted higher suppressive effects than monotherapy based on LCL-PLP, on the production of most pro-angiogenic and pro-inflammatory proteins: G-CSF, GM-CSF, M-CSF, IL-1α, IL-1β, IL-6, MCP-1, IL-13, IL-12p40, TNF-α, eotaxin, FasL, and VEGF which were reduced significantly by 25–65%. Nevertheless, LCL-DOX inhibited statistically significant (by 40–60%) the expression of proteins involved in the anti-tumor response: TIMP-1, TIMP-2, IFN-γ, MIG, PF-4, and IL-12p70 (Figure 3 and Table 1). Interestingly, combined liposomal drug therapy affected strongly (by 50–90%) the production of all pro-angiogenic and pro-inflammatory proteins as well as the levels of the antitumor proteins, IL-12p70, PF-4, and IFN-γ (Figure 3 and Table 1). Only the production of TIMP-1 was not affected by this treatment and the levels of TIMP-2 and MIG were slightly reduced (by 25%) by the combined liposomal drug therapy (Figure 3 and Table 1).

### 2.4. Combined Therapy Induced Slight Reduction of the Intratumor Oxidative Stress

**Figure 4:**
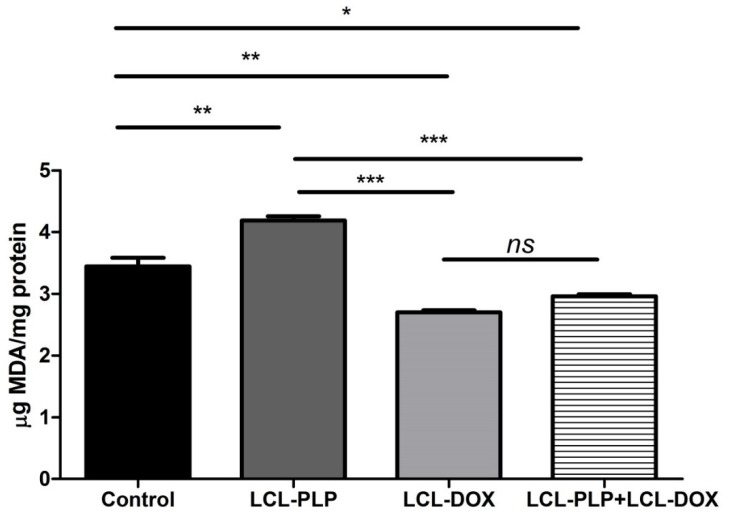
Evaluation of LCL-PLP + LCL-DOX effect on oxidative stress in tumor tissue. MDA amount was determined by HPLC analysis. The results are expressed as mean ± SD of two independent measurements. ns—not significant (*p* > 0.05); * *p* < 0.05; ** *p* < 0.01; *** *p* < 0.001.

As several studies have reported the involvement of intracellular oxidative stress in melanoma initiation and progression [16,17], we investigated whether the combined treatment could affect the physiological production of reactive oxygen species (ROS) in melanoma tumor tissue. To this purpose, the levels of MDA—a general marker for oxidative stress, were determined in tumor tissue lysates by HPLC and shown in Figure 4. Data were expressed as µmoles of MDA/mg of protein. Our data showed that LCL-PLP increased the levels of MDA by 20% compared to untreated tumors. By contrast, LCL-DOX administered alone or in combination with LCL-PLP induced a slight decrease (by 15–20%) in the intratumor level of MDA (Figure 4). In conclusion, although the reduction in the oxidative stress in tumor tissue by LCL-DOX in the combined therapy is slight, it might have contributed to the better antitumor effect of this therapy compared to each individual liposomal formulation as many tumorigenic factors are regulated by oxygen radicals [18].

### 2.5. Combined Liposomal Drug Therapy Reduced the Aggressiveness of B16.F10 Melanoma in Vivo

**Figure 5:**
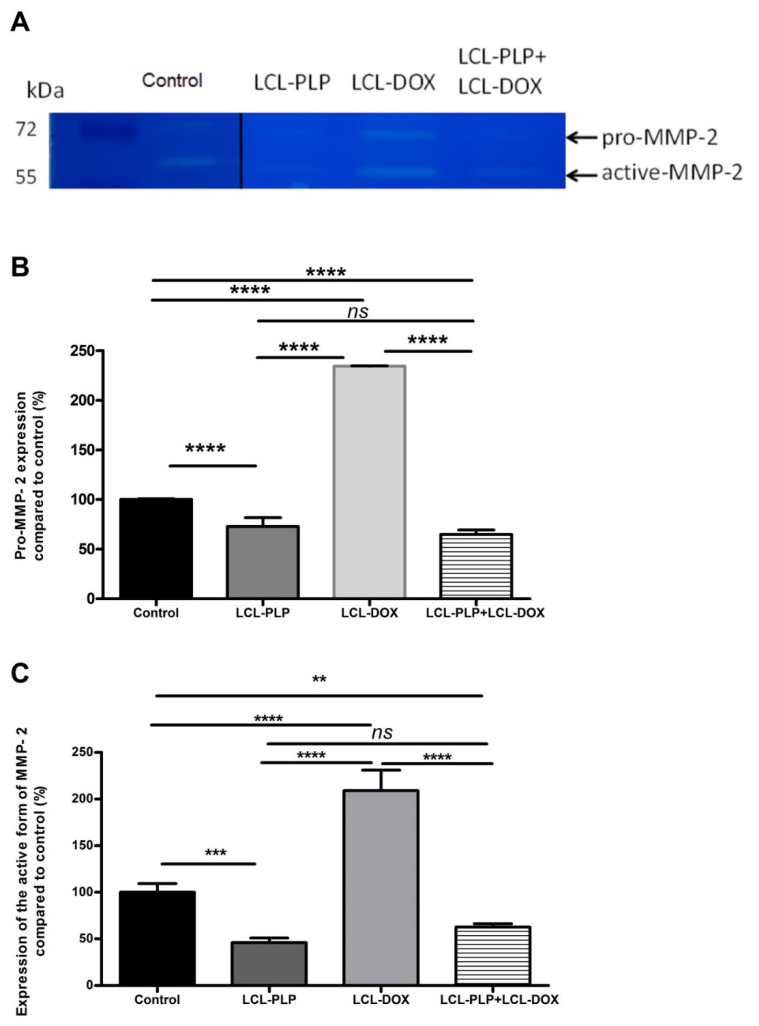
The effects of LCL-PLP + LCL-DOX on the activity of MMP-2 in tumor tissue. (**A**). Gelatin zymography gel; (**B**). Quantification of pro-MMP-2 bands in zymograms; (**C**). Quantification of active MMP-2 bands in zymograms; the results represent the mean of percentage MMP-2 activity of duplicate measurements ± SD. ns—not significant (*p* > 0.05); ** *p* < 0.01; *** *p* < 0.001; **** *p* < 0.0001.

To investigate whether the aggressiveness of B16.F10 melanoma tumors was affected by the treatments, the activity of MMP-2, a key player in tumor invasion and metastasis [19,20] was assessed in the tumor tissue lysates via gelatin zymography (Figure 5A–C).

**Figure 6:**
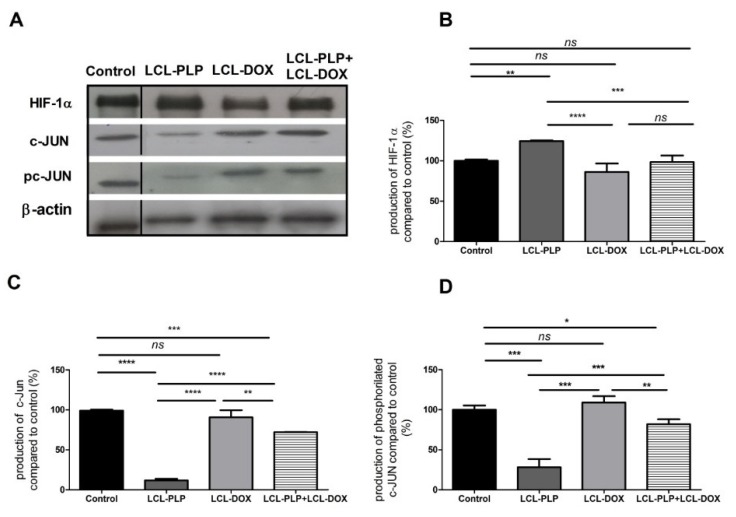
The production of HIF-1α and c-Jun proteins in tumor tissue after different treatments. (**A**). Western blot analysis of the HIF-1α and c-Jun production after different treatments. β-actin was used as loading control; (**B**). Percentages of the amount of HIF-1α protein in tumors; (**C**). Percentages of the amount of c-Jun protein in tumors; (**D**). Percentages of the amount of pc-Jun protein in tumors. The results are compared to the proteins levels in control lysates and expressed as mean ± SD of two independent measurements. ns—not significant; * *p* < 0.05; ** *p* < 0.01; *** *p* < 0.001; **** *p* < 0.0001.

Moreover, the intratumor production of the transcription factor AP-1 (c-Jun subunit) that regulates the expression of MMP-2 [21] as well as the levels of HIF-1 (α subunit, HIF-1α), that are tightly linked to the aggressive phenotype of melanoma cells [22], were determined via Western blot (Figure 6A–D).

Our data suggested that each single liposomal drug enhanced the aggressiveness of melanoma tumors. More specifically, LCL-PLP exerted suppressive actions on the production of MMP-2 (25% reduction of the zymogen form of MMP-2 (*p* < 0.0001) and 55% reduction of the active form (*p* < 0.001)) while LCL-DOX doubled the levels of the zymogen as well as the active form of MMP-2 in melanoma in vivo (Figure 5A–C). In tight connection, the production of both total and phosphorylated (active) c-Jun subunit of the transcription factor AP-1 involved in the regulation of MMP-2 expression was also reduced by LCL-PLP (70% reduction, (*p* < 0.001)) (Figure 6A,D). The reduced production as well as the activation of c-JUN protein induced by LCL-PLP in the combined therapy has the potential to decrease the metastatic potential of the tumor cells via the inhibition of MMP-2 activation. Nevertheless, LCL-PLP increased by 25% (*p* < 0.01) the intratumor expression of HIF-1α (*p* < 0.01) (Figure 6A,B). Importantly, administration of the combined liposomal therapy in B16.F10 murine melanoma-bearing mice counteracted the stimulatory effect of each single liposomal drug therapy. Thus, intratumor production of HIF-1α was not affected by this treatment (Figure 6A,B) and the levels of pro-form and active form of MMP-2 were reduced by 35–40% (*p* < 0.01) (Figure 5A–C). Moreover, the beneficial effect of LCL-PLP on the phosphorylated c-Jun expression was still seen in the combined therapy but with less extent than after the administration of LCL-PLP alone (20% reduction in phosphorylated c-Jun expression after LCL-PLP + LCL-DOX (*p* < 0.01) compared to a 70% reduction in phosphorylated c-Jun expression after LCL-PLP (*p* < 0.001) (Figure 6A,D). The better anti-tumor effect exerted by the combined liposomal therapy compared with each liposomal formulation administered individually could be explained by the dissimilar effect of the two drugs on the production of HIF-1α and MMP-2. Hence, LCL-DOX might have counteracted the stimulatory effect of the LCL-PLP on the production of HIF-1α by its slight anti-oxidant activity (Figure 4). On the other hand, LCL-PLP inhibited the activation of MMP-2 also in the presence of LCL-DOX.

### 2.6. Combination Therapy Reduced Macrophage Density in Melanoma Microenvironment

**Figure 7:**
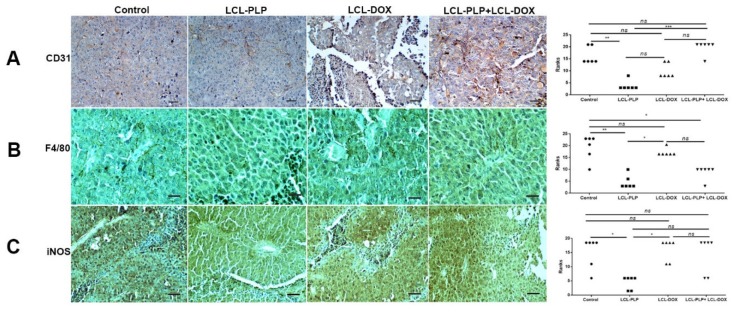
Immunohistochemical analysis of the effects of different treatments on B16.F10 murine melanoma microenvironment in vivo. (**A**) CD31 was used as a marker for proliferating endothelial cells; (**B**) F4/80 was used as a general marker for macrophages; (**C**) iNOS was used as a marker for M1 polarized macrophages. Positively stained cells appear in brown; Scale bar = 50 µm. The scores for immunoreaction intensities of tumor sections for each marker after different treatments were analyzed by using rank-based nonparametric Kruskall-Wallis test with Dunn’s test for multiple comparisons.

**Figure 8:**
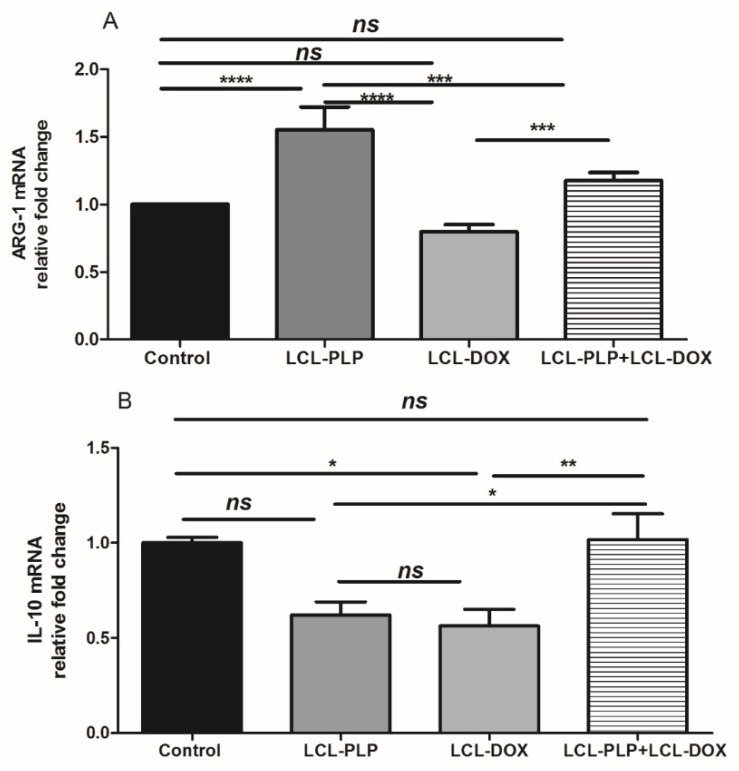
Relative quantification of ARG-1 and IL-10 mRNA expression in tumor tissue following different treatments. mRNA was quantified by RT-qPCR and the results are expressed as fold change based on the Ct calculations. (**A**). relative fold change of ARG-1 mRNA; (**B**). relative fold change of IL-10 mRNA; ns—not significant (*p* > 0.05); * *p* < 0.05; ** *p* < 0.01; *** *p* < 0.001; **** *p* < 0.0001.

To investigate whether different treatments could affect the density and the polarization of the cell types belonging to the TME, immunohistochemical analysis as well as RT-qPCR of tumors was performed and the results were shown in Figure 7 and Figure 8. Thus, tumors were evaluated for the expression of CD31 as a marker for proliferating endothelial cells [23], F4/80 as a marker for intratumor murine macrophages [24], as well as for the expression of iNOS as a marker for M1 antitumor macrophages. Moreover, the levels of IL-10 and ARG-1 mRNA in tumor tissue as markers for M2 protumor macrophages were assessed by RT-qPCR [25]. Our data suggested that only LCL-PLP reduced the expression of CD31 in tumor tissue (Figure 7A) suggesting that only this treatment might affect significantly the involvement of endothelial cells in tumor angiogenesis.

With regard to F4/80 expression on the surface of intratumor macrophages, our data suggested that a smaller number of macrophages infiltrated in tumors treated with LCL-PLP administered alone as well as in combination with LCL-DOX than in control tumors or in tumors treated with LCL-DOX alone (Figure 7B). Nevertheless, it seems that intratumor macrophages from tumors treated only with LCL-PLP skewed the polarization of the macrophages toward their protumor phenotypes, as ARG expression in these cells was strongly increased (by 50%, *p* < 0.0001) (Figure 8A) and the degree of immunoreactive macrophages for iNOS was decreased significantly (*p* < 0.05) (Figure 7C) compared with their expression in controls.

Notably, our data suggested that the immunosuppressive effect of LCL-PLP on intratumor macrophages was counteracted when LCL-PLP was administered in combination with LCL-DOX (Figure 7C and Figure 8A,B). Thus, the expression of any markers for macrophage phenotypes was not affected by the combined therapy. Altogether, these results suggested that only the number of macrophages was affected by the combined liposomal drug therapy. This finding might be linked to the anti-angiogenic action of this therapy (Figure 3 and Table 1), as TAMs are known to support tumor angiogenesis [26].

## 3. Discussion

Over the past decade, increasing evidence has suggested the role of the interaction between tumor cells and cells belonging to the TME in the modulation of the tumor cell response to different therapies [9,27]. Moreover, several key tumorigenic processes such as tumor cell proliferation, angiogenesis, immune evasion, and metastasis have been shown to be mediated by TAMs, the most abundant immune cells infiltrated in the tumor tissue [5,28]. Once recruited at the tumor site, TAMs are reeducated with the help of cytokines of the TME becoming protumor TAMs, which support the tumor growth as well as the resistance to different therapies [4,9]. Thus, our previous data have demonstrated that TAMs promoted B16.F10 melanoma growth via supporting melanoma angiogenesis and oxidative stress [11,29]. Notably, in other types of cancer, TAMs were shown to induce tumor cell resistance to DOX therapy [30,31], and cytotoxicity of liposomal DOX on human metastatic melanoma evaluated in several clinical studies did not induce any significant response on this cancer type [12,13,32]. Moreover, our earlier studies have demonstrated that LCL-PLP exerted antitumor action on B16.F10 melanoma in vivo via the inhibition of TAMs-mediated melanoma angiogenesis [11]. Based on these data, the aim of the present work was to investigate whether the beneficial action of LCL-PLP on TAMs might be exploited for the sensitization of B16.F10 melanoma—bearing mice to the treatment with LCL-DOX when they were co-administered. Therefore, in vitro cytotoxicity of the combined liposomal drug therapy on B16.F10 melanoma cells co-cultured with TAMs (Figure 1), as well as the antitumor activity of this treatment on B16.F10 murine melanoma-bearing mice (Figure 2A–F), was evaluated. Both in vitro and in vivo data demonstrated that LCL-PLP enhanced the antitumor action of LCL-DOX on B16.F10 melanoma models when they were administered in combination (Figure 1 and Figure 2A–F). Thus, combined therapy based on the simultaneous administration of LCL-PLP and LCL-DOX was superior to each single liposomal drug therapy with regard to the in vitro antiproliferative effects on these cancer cells (Figure 1), as well as the melanoma growth inhibition in vivo (Figure 2A–F). This antitumor activity could be clearly linked to the passive tumor targeting of this liposomal formulations [11,33] that enabled both drugs accumulation into the melanoma tissue. To gain more insight into the in vivo mechanisms by which LCL-PLP could enhance LCL-DOX antitumor efficacy on B16.F10 melanoma, the effects of the combined therapy on the processes responsible for melanoma progression such as angiogenesis, oxidative stress, invasion and metastasis, were assessed.

Our data suggested that the anti-angiogenic action of the combined therapy might be essential for the antitumor activity of the combined liposomal drug therapy on melanoma tumors (Figure 3 and Table 1). The high amplitude of this antitumor effect could be clearly associated with the orchestrated actions of liposomal formulations on the intratumor production of key players in tumor angiogenesis such as VEGF and bFGF (Figure 3 and Table 1). Thus, in accordance with earlier studies [34,35], LCL-DOX strongly inhibited the production of VEGF, while LCL-PLP strongly reduced the intratumor production of bFGF [11,36]. Thus, the combined administration of LCL-PLP and LCL-DOX exerted complementary suppressive action on the production of both angiogenic factors in tumor tissue (65% reduction for bFGF levels (*p* < 0.0001) and 89% reduction for VEGF levels (*p* < 0.0001)) compared with their control tumor expression (Table 1). This finding might also be explained by the reduced number of infiltrated TAMs in TME (Figure 7B) after combined therapy administration since TAMs are the most important stromal cells for the production of pro-angiogenic molecules in TME [11]. Thus, immunohistochemical analysis of TME after treatments were applied revealed that only the macrophages infiltration in melanoma microenvironment was affected by the administration of LCL-PLP + LCL-DOX (Figure 7), while the frequency of the M1 and M2 phenotypes of macrophages as well as the number of endothelial cells in TME were similar as in control tumors (Figure 7 and Figure 8A,B). It is known that high infiltration of macrophages in melanoma as well as in other tumors was previously associated with a poor prognosis [37,38]. This reducing effect on TAMs infiltration in tumors could also be mediated through the inhibition of the production of chemotactic cytokines and growth factors (such as M-CSF and MCP-1) mediating monocytes recruitment and differentiation [39,40] which were reduced in tumors treated with LCL-DOX in the presence of LCL-PLP.

To prove that LCL-PLP + LCL-DOX treatment was superior to single liposomal drug therapy with regard to the antitumor efficacy on melanoma development, the aggressiveness of this tumor after the combined therapy was evaluated. Therefore, the intratumor activity of MMP-2 that is involved in tumor progression and metastasis [41] was assessed using gelatin zymography (Figure 5A–C). As previously described [42], DOX encapsulated in LCL administered alone acted as an enhancer of MMP-2 activity (Figure 5A–C). Notably, as inhibitor of MMP-2 activity in tumor tissue [43], PLP encapsulated in LCL overcame the protumor action of DOX when they were administered as combined liposomal drug therapy in B16.F10 melanoma-bearing mice (Figure 5A–C). Thus, when the liposomal drugs were administered concurrently, the activity of MMP-2 was reduced by 35%–40% (*p* < 0.01) (Figure 5A–C) compared to control tumor activities of this enzyme. An explanation for this finding might be given by the similar action of the LCL-PLP + LCL-DOX on the intratumor production of the active c-Jun subunit of transcription factor AP-1 (Figure 6A,D) that regulates the expression of MMP-2 [21]. Interestingly, our data suggested that not only monotherapy based on LCL-DOX, but also that based on LCL-PLP, could induce an aggressive phenotype of this cancer type. Thus, our data demonstrated that LCL-PLP administered alone exerted stimulatory action on the levels of the subunit α of the transcription factor HIF-1 (Figure 6A,B) that is constitutively expressed in aggressive melanoma cells [44,45]. This effect is a consequence of the glucocorticoid-mediated degradation of the Von Hippel Lindau protein involved in polyubiquitylation of HIF-1α and finally its degradation [46]. Notably, LCL-DOX administered concomitantly with LCL-PLP counteracted this protumor action of the liposomal glucorticoid formulation (Figure 6A,B). Nevertheless, the combined therapy was not able to suppress the intratumor production of HIF-1α (Figure 6A,B). This limitation might also be linked to the slight anti-oxidant action of the combined therapy (Figure 4) that did not affect sufficiently ROS levels responsible for the suppression of the prolyl hydroxylases—enzymes which are involved in HIF-1α degradation [18]. Another limitation of this therapy is related to the immunosuppressive effects induced by PLP-induced expression of PD-1, a negative regulator of T cell activation [47]. However, recently published data have indicated that DOX inhibited the expression of PD-L1 on cancer cells [48,49].

Altogether, our data demonstrated that LCL-PLP was able to sensitize B16.F10 murine melanoma to LCL-DOX and to enhance therapeutic outcome of this tumor-targeted therapy. Thus, the antitumor activity of the combined liposomal drug treatment in melanoma in vivo was mainly based on the suppression of tumor angiogenesis in TME as a result of a reduction in the intratumor macrophage density. Nonetheless, the suppression of metastatic and invasive capacity of the melanoma cells might also contribute substantially to the high amplitude of the antitumor efficacy of this therapy.

## 4. Materials and Methods

### 4.1. Preparation of Liposomal Formulations

LCL-PLP and LCL-DOX were prepared by lipid film hydration method as previously described [11] To encapsulate DOX in LCL a liposomal transmembrane proton gradient with ammonium sulfate was created as described by Bolotin et al. [50]. Thus, the lipid film was prepared as for the LCL-PLP but it has been rehydrated with a buffer containing 240 mM (NH_4_)_2_SO_4_ and 1M Titriplex. After extrusion, free (NH_4_)_2_SO_4_ was removed by dialysis against a buffer (HBS) containing: 20mM HEPES (pH 7.5), 0.8% NaCl and 1mM Titriplex. After dialysis, liposomes were mixed with 4 mg/ml DOX solution in HBS buffer at a volume ratio of 1/1. The mixture was incubated for 1h at 60 °C in a water bath. To remove unencapsulated DOX, the suspension was further dialyzed in HBS buffer.

### 4.2. Cell Types and Culture Conditions

B16.F10 murine melanoma cells (ATCC, CRL-6475) were cultured in DMEM (Lonza) as described previously [51]. For proliferation assay, B16.F10 melanoma cells were co-cultured with bone marrow-derived macrophages (BMDMs) isolated from the femurs of male C57BL/6 mice (Cantacuzino Institute, Bucharest, Romania) as we previously reported [52] at a cell density ratio of 1:4 [14].

### 4.3. In Vivo Melanoma Model

In vivo melanoma tumors were induced by subcutaneous inoculation of 10^6^ B16.F10 cells in the right flank of 6–8 weeks-old male C57Bl/6 mice (Cantacuzino Institute, Bucharest, Romania). Treatments started at day 11 after cell inoculation when tumors were about 100 mm^3^. Experiments were performed according to the national regulations and were approved by the Babes-Bolyai University Ethics Committee (Cluj-Napoca, Romania, Project ID: PN-III-P4-ID-PCE-2016–0342 No.91/2017, Approval no. 4335/19.03.2018).

### 4.4. Evaluation of the Antiproliferative Activity of the Liposomal Combination Therapy on B16.F10 Cells

To determine the effects of different liposomal treatments on tumor cell proliferation, B16.F10 melanoma cells (1000 cells/well) together with BMDMs (4000 cells/well) were seeded in a 96-well plate for 24 h. Different concentrations of DOX encapsulated in LCL (ranging from 0.005–0.37 µM) were administered alone or in combination with 410 µM LCL-PLP and tested in triplicate to assess the IC50 values. Cells incubated only with medium were used as control. Proliferative activity of the cells at 48h after treatment administration was tested by using ELISA BrdU-colorimetric immunoassay (Roche Applied Science, Penzberg, Germany) according to the manufacturer’s instructions [51]. This method is based on the incorporation of the pyrimidine analogue—bromodeoxyuridine (BrdU)—instead of thymidine into the DNA of proliferating cells. B16.F10 melanoma cells in the co-culture model were incubated with BrdU solution for 24 h and the culture medium was completely removed from each well. Following this step, the cells were fixed and the DNA was denatured. A monoclonal antibody conjugated with peroxidase—anti-BrdU-POD—was added in each well, in order to detect the incorporated BrdU in the newly synthesized cellular DNA. The antibody was removed after 1 h incubation, and the cells were washed three times with phosphate buffered saline. A peroxidase substrate (tetramethyl-benzidine) was added in each well, and the immune complexes were detected by measuring the absorbance of the reaction product at 450 nm with a reference wavelength of 655 nm.

### 4.5. Effects of Combination Therapy (LCL-PLP + LCL-DOX) on Tumor Growth

LCL-PLP (10 mg/kg) and LCL-DOX (5 mg/kg) were administered i.v simultaneously as well as separately at days 11 and 14 after tumor cell inoculation (*n* = 5 mice/group). In the present study, we used the dose of 10 mg/kg of LCL-PLP in order to reduce the potential side effects noticed at the previous administered dose of 20 mg/kg (body weight loss, weight loss of spleen and liver) associated with glucocorticoid administration [11] and to increase the dose of the cytotoxic agent since PLP is used only as a modulator of the tumor microenvironment, not as an antitumor agent. Two different doses of LCL-DOX (2 mg/kg and 5 mg/kg) were tested but no significant effect of 2 mg/kg LCL-DOX on the tumor growth was noticed. Mice treated with empty LCL were used as controls. Body weight and tumor size of mice was monitored daily during treatments. None of the above-mentioned side effects were noticed at any of the experimental groups. Tumor volume was determined according to the formula: V = 0.52a^2^b, where a is the smallest and b is the largest superficial diameter. On day 15, mice were sacrificed and tumors were collected.

### 4.6. Western Blot Quantification of the HIF-1α and c-Jun Levels in Tumor Tissue

Tumors isolated from each experimental group were weighted and then pooled to obtain whole tissue lysates, and Western blot analysis for HIF-1α and c-Jun was performed as reported [29,53]. 10 µg of total protein was loaded per lane onto a 10% polyacrylamide gel. Rabbit mAb anti-mouse HIF-1α (Abcam, Newcastle, UK) and rabbit polyclonal antibodies specific for total as well as phosphorylated mouse c-Jun (Santa Cruz Biotechnology, Dallas, USA), were diluted 500-fold. The antibody against mouse β-actin (Sigma-Aldrich; Germany) was diluted 1000-fold.

### 4.7. Protein Array Analysis of the Inflammatory/Angiogenic Protein Levels in Tumors

To assess whether the combination therapy alters the cell production of proteins involved in angiogenesis and inflammation, a screening for 24 proteins involved in angiogenesis and inflammation was performed as described previously [51] using a protein array of RayBio^®^ Mouse Angiogenic protein Antibody Array membranes 1.1 (RayBiotech Inc., Peachtree Corners, GA, USA). Briefly, one array membrane containing 24 types of primary antibodies against specific mouse proteins was used per experimental group. The array membranes were incubated with 250 µg of proteins of tissue lysates, overnight at 4 °C. A mixture of secondary biotin-conjugated antibodies against the same angiogenic factors as those for primary antibodies, was added on the membranes and incubated for 2 h at room temperature, followed by incubation with HRP-conjugated streptavidin for additional 2 h. Each incubation step was followed by five washing steps. Thereafter, the membranes were incubated with a mixture of two detection buffers for 1 min, exposed to an X-ray film (Kodak) for 4 min and then the films were developed. The protein expression level was quantified by measuring the intensity of the color of each spot on the membranes, in comparison to the positive control spots already bound to the membranes, using TotalLab Quant Software version 12 for Windows. Each protein level in the lysates of the treated groups was expressed as percentage of the same protein level in the lysates from untreated mice (control group). The level of each protein from each experimental group was determined through a duplicate.

### 4.8. HPLC Determination of Malondialdehyde Levels in Tumor Cell Lysates

Malondialdehyde (MDA) levels in tumor tissue lysates were determined according to the method employed by Karatas et al. (2002) through HPLC [51].

### 4.9. RT-qPCR Determination of Arginase-1 and IL-10 Expression

The relative expression of mRNA for Arginase-1 and IL-10 in tumor tissues was determined by RT-qPCR as previously reported [14]. In brief, total RNA was isolated from tumor tissue of each experimental group using an RNA kit (peqGOLD Total RNA Kit, PeqLab). To avoid contamination with genomic DNA, 1 µg of total RNA was digested with 1U of RNase free DNase (Thermo Scientific) for 30 min at 37 °C, followed by the addition of EDTA and incubation at 65 °C for 10 min. After DNase digestion, 750 ng of total RNA was revers-transcribed into cDNA with Verso cDNA kit (ThermoScientific) according to the manufacturer’s instructions. Identical samples from each experimental group were processed in the absence of reverse transcriptase and served as controls for genomic DNA contamination. Reverse transcription products (1 μL) were amplified in a 25-μL reaction mix containing 1 × Maxima SYBR Green qPCR Master Mix (Thermo Scientific), and 0.3 μM of each primer using a Corbett RotorGene instrument using the following cycling parameters: preincubation at 95 °C for 10 min, then cycling: 95 °C for 15 s, 60 °C for 30 s, and then 72 °C for 30 s. To check for the primers specificity, melting curves were generated. The primers used for gene amplification are shown in Table 2. Gene expression was calculated by relative quantitation using the comparative Ct method (ΔΔCt), as previously described [54].

### 4.10. Determination of MMP-2 Activity by Gelatin Zymography

The assay was performed following a published protocol [55]. Thus, 30 µg of total proteins were loaded on a 7.5% acrylamide gel containing 1 mg/mL gelatin and separated by electrophoresis under nonreducing conditions. Subsequently, gels were washed two times for 30 min at room temperature with a buffer containing 2.5% Triton X-100, 50mM Tris-HCl (pH 7.5), 5mM CaCl_2_, and 1 µM ZnCl_2_. Then, gels were incubated overnight at 37 °C in a buffer containing 1% Triton X-100, 50mM Tris-HCl (pH 7.5), 5mM CaCl_2_, and 1µM ZnCl_2_. The next day, the gels were stained with Coomassie blue staining solution for 1 h, followed by distaining until the bands became visible. The area of the enzyme activity was determined by densitometry analysis using Image J software. The experiment was repeated twice.

### 4.11. Immunohistochemistry Analysis of Tumor Tissue

Immunohistochemical analysis for CD31, NOS2 and F4/80 was performed as previously described [53]. The primary antibodies were diluted as follows: anti-CD31 antibody (rabbit IgG anti-mouse CD31, Abcam, ab124432) was diluted 1000-fold, anti-NOS2 antibody (mouse IgG anti-mouse NOS2, Santa Cruz Biotechnology INC, sc-7271) was diluted 500-fold and for F4/80 mouse macrophage receptor the antibody (rat IgG anti-mouse F4/80, Bio-Rad, MCA497) was diluted 250-fold. We used the following scoring system to evaluate the area (%) of positive immunoreaction: 0.5: 5%–20%; 1: 20%–40%; 2: 40%–60%; 3: 60%–80%; 4: 80%–100%.

### 4.12. Statistical Analysis

Data from different experiments were reported as mean ± standard deviation (SD). To analyze the treatment effects on the levels of angiogenic/inflammatory proteins in cells, a two-way ANOVA with Bonferroni correction for multiple comparisons was used. The effects of different treatments on tumor growth as well as on the on the mean production of different key markers for different protumor processes were analyzed using a one-way ANOVA with Bonferroni correction for multiple comparisons. The scores for the immunoreaction intensities of tumor sections after different treatments were analyzed using a rank-based nonparametric Kruskall–Wallis test with Dunn’s test for multiple comparisons. All statistical analyses were performed by using GraphPad Prism version 6 for Windows, GraphPad Software (San Diego, CA, USA). The IC50 values of different treatments were calculated using a non-linear regression of sigmoidal dose response curves offered by the software mentioned above.

## Figures and Tables

**Table 1:** Effects of the liposomal therapies on the intratumor production of angiogenic and inflammatory proteins.

Angiogenic/Inflammatory Proteins	Percentage of Reduction (−)/Increase (+) in Intratumor Production of Proteins Involved in Tumor Angiogenesis/Inflammation Following Different Treatments Compared Their Levels in Control Tumors		
	LCL−PLP	LCL−DOX	LCL−PLP + LCL−DOX
Granulocyte-colony stimulating factor (G-CSF)	−5.96 ± 4.03(*ns*)	−27.52 ± 3.93(**)	−49.73 ± 2.68(****)
Granulocyte−macrophage-colony stimulating factor (GM-CSF)	−16.80 ± 12.03 (*ns*)	−42.57 ± 4.35 (****)	−73.93 ± 0.94(****)
Monocyte−colony stimulating factor (M-CSF)	−30.00 ± 23.88(**)	−42.54 ± 2.81 (****)	−65.54 ± 4.04(****)
Insulin growth factor II (IGF-II)	−8.15 ± 24.81(*ns*)	−15.67 ± 2.14 (*ns*)	−70.12 ± 1.96 (***)
Interleukin 1α (IL-1α)	−15.52 ± 14.06(*ns*)	−36.20 ± 0.74(****)	−55.02 ± 1.93(****)
Interleukin 1β (IL-1β)	−48.17 ± 14.25(****)	−35.07 ± 3.80(***)	−53.58 ± 1.16(****)
Interleukin 6 (IL-6)	−26.49 ± 4.67(**)	−39.77 ± 7.08(****)	−52.05 ± 6.81(****)
Interleukin 9 (IL-9)	−42.70 ± 3.83(****)	−10.81 ± 1.53(*ns*)	−48.24 ± 5.69(****)
Interleukin 12 p40 (IL 12-p40)	−31.33 ± 0.35(***)	−63.90 ± 2.76(****)	−80.16 ± 0.07(****)
Interleukin 13 (IL-13)	+9.84 ± 5.91(*ns*)	−28.10 ± 1.29(***)	−51.88 ± 4.43(****)
Tumor necrosis factor α (TNF-α)	−12.42 ± 33.15(*ns*)	−64.83 ± 6.68(****)	−65.47 ± 0.00(****)
Monocyte chemoattractant protein-1 (MCP-1)	−32.62 ± 4.46(***)	−25.40 ± 5.37(*)	−69.89 ± 1.95(****)
Eotaxin	−64.09 ± 48.39(****)	−67.59 ± 1.33(****)	−58.79 ± 1.17(****)
Fas ligand (FasL)	−76.62 ± 17.48(****)	−57.25 ± 0.00(****)	−57.98 ± 5.15(****)
Basic fibroblast growth factor (bFGF)	−87.15 ± 4.06(****)	−25.44 ± 9.35(*)	−64.86 ± 0.60(****)
Vascular endothelial growth factor (VEGF)	+5.32 ± 63.80(*ns*)	−64.91 ± 19.15(****)	−89.14 ± 12.10(****)
Leptin	−4.78 ± 6.36(*ns*)	−40.51 ± 4.45(****)	−60.75 ± 15.09(****)
Thrombopoietin (TPO)	+19.46 ± 3.42(*ns*)	−20.31 ± 13.03(*ns*)	−78.09 ± 2.80(****)
Tissue inhibitor of matrix metalloproteinase 1 (TIMP-1)	−2.52 ± 10.78(*ns*)	−52.59 ± 0.87(****)	+3.76 ± (*ns*)
Tissue inhibitor of matrix metalloproteinase 2 (TIMP-2)	−10.02 ± 10.57(*ns*)	−42.47 ± 19.90(****)	−24.96 ± 21.18(*)
Platelet factor 4 (PF4)	−15.18 ± 2.11(*ns*)	−57.68 ± 3.72(****)	−58.17 ± 8.63(****)
Interleukin 12 p70 (IL-12p70)	−10.02 ± 10.57(*ns*)	−46.74 ± 1.08(****)	−63.45 ± 2.07(****)
Interferon γ (IFN-γ)	−4.69 ± 0.42(*ns*)	−45.92 ± 4.58(****)	−57.69 ± 7.82(****)
Monokine induced by IFN-γ (MIG)	−15.82 ± 1.25(*ns*)	−42.85 ± 33.18(****)	−26.30 ± 37.68(**)

The results represent the mean ± SD of two independent measurements. LCL-PLP, percentages of reduction or increase in different protein production in tumors treated with 10 mg/kg LCL-PLP compared with their production in untreated tumors; LCL-DOX, percentages of reduction or increase in different protein production in tumors treated with 5 mg/kg LCL-DOX compared with their production in untreated tumors; LCL-PLP + LCL-DOX, percentages of reduction or increase in different protein production in tumors treated with 10 mg/kg LCL-PLP and 5 mg/kg LCL-DOX compared with their production in untreated tumors. *p* value was determined to evaluate statistical significance of the data and was calculated by Two-way ANOVA analysis with Bonferroni posttest (ns, not significant, *p* >0.05; * *p* < 0.05; ** *p* < 0.01*** *p* < 0.001, **** *p* < 0.0001).

**Table 2:** Primer sets used for RT-qPCR.

Name of Genes	Forward Primer (5′-3′)	Reverse Primer(5′-3′)
Mouse β-actin	TCTTTGCAGCTCCTTCGTTGCCGGTCC	GTCCTTCTGACCCATTCCCACCATCACAC
Mouse Arg-1	CTCCAAGCCAAAGTCCTTAGAG	AGGAGCTGTCATTAGGGACATC
Mouse IL-10	GGTTGCCAAGCCTTATCGGA	ACCTGCTCCACTGCCTTGCT

## Abbreviations

ANOVA	Analysis of variance
Arg-1	arginase-1
bFGF BMDMs	Basic fibroblast growth factor Bone marrow-derived macrophages;
BrdU	bromodeoxyuridine
DMEM	Dulbecco’s Modified Eagle’s medium
DOX	doxorubicin
FasL	Fas ligand
G-CSF	Granulocyte-colony stimulating factor
GM-CSF	Granulocyte-macrophage-colony stimulating factor
HPLC	high-performance liquid chromatography
IC50	Half maximal inhibitory concentration
IFN-γ	Interferon γ
IGF-II	Insulin growth factor II
IL-1α	Interleukin 1α
IL-1β	Interleukin 1β
IL-4	Interleukin 4
IL-6	Interleukin 6
IL-9	Interleukin 9
IL-10	Interleukin 10
IL-12 p40	Interleukin 12 p40
IL-12 p70	Interleukin 12 p70
IL-13	Interleukin 13
MDA	Malondialdehyde
MIG	Monokine induced by IFN-γ
M-CSF	Monocyte-colony stimulating factor
MCP-1	Monocyte chemoattractant protein-1
PF-4	Platelet factor 4
PLP	Prednisolone disodium phosphate
SD	Standard deviation
TAMs	tumor - associated macrophages
TBS	Tris-buffered saline
TIMP-1	Tissue inhibitor of metalloproteinase 1
TIMP-2	Tissue inhibitor of metalloproteinase 2
TME	Tumor microenvironment
TNF-α	Tumor necrosis factor α
TPO	Thrombopoietin
VEGF	Vascular endothelial growth factor
